# Exposure to Indoor Particulate Matter Worsens the Symptoms and Acute Exacerbations in Chronic Obstructive Pulmonary Disease Patients of Southwestern Taiwan: A Pilot Study

**DOI:** 10.3390/ijerph14010004

**Published:** 2016-12-22

**Authors:** Miao-Ching Chi, Su-Er Guo, Su-Lun Hwang, Chiang-Ting Chou, Chieh-Mo Lin, Yu-Ching Lin

**Affiliations:** 1 Chronic Diseases and Health Promotion Research Center, Chang Gung University of Science and Technology (CGUST), Puzi City 613, Taiwan; mcchi@mail.cgust.edu.tw (M.-C.C.); slhuang@mail.cgust.edu.tw (S.-L.H.); ctchou@gw.cgust.edu.tw (C.-T.C.); 2 Department of Respiratory Care, Chang Gung University of Science and Technology, Puzi City 613, Taiwan; f124510714@adm.cgmh.org (C.-M.L.); lin0927@adm.cgmh.org.tw (Y.-C.L.); 3 Department of Nursing, Chang Gung University of Science and Technology, Chiayi Campus, Puzi City 613, Taiwan; 4 Division of Pulmonary and Critical Care Medicine, Chang Gung Memorial Hospital, Puzi City 613, Taiwan; 5 Graduate Institute of Clinical Medical Sciences, College of Medicine, Chang Gung University, Taoyuan 333, Taiwan; 6 Department of Respiratory Care, Chang Gung University, Taoyuan 333, Taiwan

**Keywords:** indoor air quality, COPD assessment test, acute exacerbation of COPD (AECOPD), hospitalization, respiratory symptoms, chronic obstructive pulmonary disease

## Abstract

Ambient particulate matter (PM) can trigger adverse reactions in the respiratory system, but less is known about the effect of indoor PM. In this longitudinal study, we investigated the relationships between indoor PM and clinical parameters in patients with moderate to very severe chronic obstructive pulmonary disease (COPD). Indoor air quality (PM_2.5_ and PM_10_ levels) was monitored in the patients’ bedroom, kitchen, living room, and front door at baseline and every two months for one year. At each home visit, the patients were asked to complete spirometry and questionnaire testing. Exacerbations were assessed by chart review and questionnaires during home visits. Generalized estimating equation (GEE) analysis (*n* = 83) showed that the level of wheezing was significantly higher in patients whose living room and kitchen had abnormal (higher than ambient air quality standards in Taiwan) PM_2.5_ and PM_10_ levels. Patients who lived in houses with abnormal outdoor PM_2.5_ levels had higher COPD Assessment Test scores (physical domain), and those who lived in houses with abnormal PM_10_ levels in the living room and kitchen had higher London Chest Activity of Daily Living scores. Increased PM levels were associated with worse respiratory symptoms and increased risk of exacerbation in patients with moderate to very severe COPD.

## 1. Introduction

Ambient particulate matter (PM) can have a negative impact on respiratory diseases [1], increasing their prevalence [2] and the number of associated hospital admissions [3]. In particular, ambient PM is directly associated with mortality rate [4] and risk of acute exacerbation in chronic obstructive pulmonary disease (COPD) [5,6,7,8]. PM_2.5_ with a mean aerodynamic diameter of ≤2.5 μm is more likely to cause respiratory diseases than PM_10_ with a mean aerodynamic diameter of ≤10 μm. PM_10_ is the most commonly associated with sea salt, road/soil dust, and construction activities, whereas PM_2.5_ is associated with transformation of gaseous species, industrial combustion processes, and forest fires [9].

Acute exacerbation (AE) is a major cause of death [10] and impaired quality of life in patients with COPD [11,12], an increased burden of medical and social resources, and increased expenditures by families and governments [13]. The probability of AE differs greatly among patients with COPD. A previous history of medically recorded treatments for patients with acute exacerbation of COPD (AECOPD) predicts the possibility of having frequent AEs (≥2 times in a year) [14]. Frequent AEs in patients with COPD induce loss of lung function [15,16], promote a decline in daily activities [17], and worsen dyspnea, ultimately causing disability [16,18]. Dominici et al. reported that elevated levels of PM_2.5_ in 204 cities of the United States were associated with a significant increase of hospital admissions in patients with COPD on the same day of exposure (lag0) and an increase of hospital admissions in patients with heart failure the next day of exposure (lag1), suggesting PM_2.5_ has a negative and immediate impact on AE in patients with COPD [3].

Although some studies have demonstrated that ambient PM is associated with morbidity, admission rate, and mortality of respiratory diseases, few studies have investigated the effects of indoor PM on the symptoms, lung function, and AEs in patients with COPD. The prevalence of respiratory diseases has been increasing worldwide during the past decade. In 2012, respiratory diseases were the third leading global cause of death worldwide [19]. The Ministry of Health and Welfare of the Republic of China indicated that in 2014, the COPD-related standardized death rate in Taiwan was 15.3 deaths per 100,000 individuals, which is lower than that in the Unites States (24.7 deaths per 100,000 individuals), but higher than that in Asian neighbors such as Japan, Singapore, and Korea [20]. In the Chiayi County of Taiwan, the standardized death rate of COPD was 16.4 deaths per 100,000 individuals, and COPD was the seventh leading cause of death (the sixth in males and the tenth in females) [21]. The purpose of this study was to investigate the relationships between PM levels at home and clinical parameters in patients with moderate to very severe COPD.

## 2. Materials and Methods

### 2.1. Study Design

A longitudinal design was used in the present study. The frequency of home visits for each participant was every two months in one year (for a total of six visits). In this study, all participants were patients with AECOPD and were recruited from outpatient clinics of the division of Pulmonary and Critical Care Medicine in a regional hospital in southwestern Taiwan from March 2014 to May 2016. This study was approved by the Ethical Committee of the Human Body of the Chang Gung Medical Foundation (102-2417B). After explaining the purpose and procedure to possible participants, informed consent was obtained from all subjects who were willing to take part in the study. After that, home visits for data collection were arranged, including interviewer-administered questionnaires and environmental data collection through observation and equipment. In addition, to avoid the preparation of meals, we made each visit between 9 a.m. and 11 a.m. or 2 p.m. and 4 p.m.

### 2.2. Participants

Inclusion criteria were as follows: (1) age ≥40 years; (2) COPD diagnosis by physicians and hospital admission of AE ≥1 time within the previous 3 months; (3) moderate to very severe COPD according to Global Initiative for Chronic Obstructive Lung Disease (GOLD) guidelines (forced expiratory volume in 1 second [FEV_1_] predicted <80%) [15]; and (4) ability to understand and communicate in Chinese or Taiwanese. We excluded patients with heart disease, asthma, tuberculosis, or cancer.

### 2.3. Measurements and Instruments

This study was designed using a structured self-reported questionnaire that was divided into four sections: demographic and clinical characteristics, respiratory symptoms, COPD Assessment Test (CAT), and London Chest Activity of Daily Living (LCADL) scale. The number of emergency room (ER) visits or hospital admissions was collected by chart review. Environmental data (air quality assessment) included PM_2.5_ and PM_10_ levels.

#### 2.3.1. Demographic and Clinical Characteristics

Demographics included age, gender, weight status, employment status, education level, exercise habits, smoking status, family smoking, living environment, disease severity, body mass index (BMI), and waist-to-hip ratio. Clinical characteristics included Charlson comorbidity index (CCI), FEV_1_ at first visit, and length of COPD diagnosis in years. FEV_1_ at every visit was considered, as a confounding variable in the analysis of the association between PM levels and the symptoms and acute exacerbations of COPD patients. The CCI is used as a measurement of the 1-year mortality risk, and includes 19 comorbidities. The CCI scores are 0 (no symptoms), 1, 2, 3, or 6 per item. Higher CCI scores indicate a higher level of burden of the disease.

#### 2.3.2. Respiratory Symptoms

Respiratory symptoms were evaluated using a modified version of the symptoms section of the St. George’s Respiratory Questionnaire (SGRQ). This section addresses the frequency of respiratory symptoms and uses a five-point Likert scale scoring for questions, with options of 0 (disagree strongly), 1 (disagree), 2 (no response), 3 (agree), and 4 (agree strongly). Higher scores indicate more respiratory problems experienced by patients. Cronbach’s alpha of the Chinese version [22] and that of the version of the present study was 0.76 and 0.78, respectively, indicating good reliability.

#### 2.3.3. COPD Assessment Test (CAT)

The CAT consists of eight items related to COPD symptoms, including cough, phlegm (mucus), chest tightness, breathlessness after walking up a hill or one flight of stairs, activity limitation at home, confidence in leaving home, sleep quality, and energy. Each item is scored from 0 to 5. A study of the Chinese version of CAT reported that the severity of lung function is associated with an increase of CAT score [23]. The Cronbach’s alpha was 0.81, evidencing that the Chinese version of the CAT had good internal consistency [23]. The CAT in the Chinese version is a simple, reliable, and validated test for patients with COPD [23].

#### 2.3.4. London Chest Activity of Daily Living (LCADL) Scale

The LCADL is divided into four categories (self-care, domestic, physical, and leisure) and consists of 17 items. Each item is scored from 0 to 5 points. It has been used to assess breathlessness during daily living activities in patients with heart diseases and COPD. Higher LCADL scores show greater limitation in daily living activities. The LCADL has good construct validity and concurrent validity with SGRQ, Extended Activities of Daily Living (EADL), the Shuttle test, and Forced Vital Capacity (FVC) [24]. It is also a measuring tool with good reliability (Chronbach’s alpha = 0.98).

#### 2.3.5. Acute Exacerbation

We used the number of emergency room (ER) visits or COPD-related hospitalizations as an objective representation of AE. The number of ER visits and admissions were counted every 2 months using chart review.

#### 2.3.6. Air Quality Assessment

Outdoor and indoor air sampling for PM_2.5_ and PM_10_ was performed by using an aerosol spectrometer (Model TSI8532) at every home visit. We detected PM_2.5_ and PM_10_ in four areas, including the front of the house (outside the front door), living room, bedroom, and kitchen. Each detection was performed three times for 1 min. The daily mean or 24-h maximum of PM_10_ is 125 μg/m^3^, and that of PM_2.5_ is 35 μg/m^3^ according to the 2012 Environment Protection Administration of Taiwan ambient air quality standards [25].

### 2.4. Statistical Analyses

Demographic and clinical characteristics are presented as number and percentage or mean and standard deviation. Due to repeated measurements (each patient was followed for a minimum of two sessions and a maximum of seven) in this study, we performed a generalized estimating equation (GEE) to account for the outcome dependency within subjects [26]. In the GEE analysis, we treated the air quality status at various areas (e.g., normal/abnormal outdoor levels of PM_2.5_) as independent variables with adjustment for control variables (age at baseline, FEV_1_ predicted at each visit, smoking status, and whether the window was open or not). Normal PM levels were defined as per Taiwan ambient air quality standards. There were two types of outcome measures: the subjective measures (i.e., CAT, LCADL) and the objective measures (i.e., the number of ER visits or admissions due to AE of COPD). We conducted data analyses using SPSS 22 (IBM Corp., Armonk, NY, USA).

## 3. Results

### 3.1. Patient Characteristics

Twenty-six participants were initially included. Seven participants were excluded because they withdrew their consent during the study period. There was no statistically significant difference between the included (*n* = 19) and excluded (*n* = 7) participants in variables such as age, gender, BMI, and lung function. In total, data from 19 participants were collected every 2 months. However, some participants did not complete the six home visits because of hospitalization, travel to another city, or death. In total, we collected data from 83 home visits. All 19 participants were male and their mean age was 72.6 ± 6.8 years. Most of them were farmers (42.1%), 57.9% were living with their children or spouses, and all of them believed in Taoism. Mean BMI was 22.7 ± 6.7, mean disease duration was 5.1 years, mean FEV_1_ at the first visit was 42.4%, and mean waist-to-hip ratio was 1.00 ± 0.11 (Table 1).

### 3.2. PM_2.5_ and PM_10_ Concentrations and Abnormal Proportions

Mean PM_2.5_ concentrations for the bedroom, kitchen, main living area, and outdoor area were 104.7 ± 66.5, 119.0 ± 74.4, 104.7 ± 66.5, and 120.6 ± 75.2 μg/m^3^, respectively. Mean PM_10_ concentrations for the bedroom, kitchen, main living area, and outdoor area were 104.6 ± 66.8, 123.4 ± 77.3, 114.6 ± 66.8, and 131.3 ± 81.3 μg/m^3^, respectively. On average, most indoor and outdoor PM_2.5_ concentrations were abnormal (i.e., >35 μg/m^3^ for PM_2.5_ and >125 μg/m^3^ for PM_10_). The abnormal proportions of the bedroom, kitchen, main living area, and outdoor area were 0.85 ± 0.17, 0.89 ± 0.19, 0.86 ± 0.16, and 0.89 ± 0.13, respectively. However, only about half of indoor and outdoor PM_10_ concentrations were abnormal. The abnormal proportions for the bedroom, kitchen, main living area, and outdoor area were 0.46 ± 0.35, 0.43 ± 0.29, 0.46 ± 0.32, and 0.54 ± 0.36, respectively.

### 3.3. Association between PM_2.5_ Concentrations, Symptoms, and Activities of Daily Living (ADL)

Table 2 shows the descriptive statistics of outcome measures according to normal or abnormal PM_2.5_ levels. GEE analysis showed that the level of wheezing was higher in patients living in houses with abnormal PM_2.5_ levels in the outdoors (*B* = 1.46, *p* < 0.01), living room (*B* = 0.80, *p* < 0.001) and kitchen (*B* = 1.03, *p* < 0.01) than in those with normal PM_2.5_ levels. Patients living in houses with abnormal outdoor PM_2.5_ levels also had higher CAT physical scores (*B* = 0.93, *p* < 0.05) than those living in houses with normal PM_2.5_ levels. However, there was no association between air quality and LCADL scores.

### 3.4. Association between PM_10_ Concentrations, Symptoms, and ADL

Table 3 shows the descriptive statistics of outcome measures according to normal or abnormal PM_10_ levels. Similar to the results of PM_2.5_, the level of wheezing was higher in patients living in houses with abnormal PM_10_ levels in the living room (*B* = 0.36, *p* < 0.05) and in the kitchen (*B* = 0.38, *p* < 0.05) than in those with normal PM_10_ levels. GEE analysis also showed that the LCADL scores were higher in patients living in houses with abnormal PM_10_ levels in the living room (*B* = 5.58, *p* < 0.05) and kitchen (*B* = 6.18, *p* < 0.05). However, there was no association between air quality and CAT scores.

### 3.5. Association between PM (PM_2.5_ and PM_10_) and Acute Exacerbation of COPD

Table 4 displays the number and rate of ER visits and admissions due to AE of COPD according to normal or abnormal PM levels, including PM_2.5_ and PM_10_. No events were recorded for normal PM_2.5_ levels. Therefore, the parameters of GEE analysis could not be converged. For PM_10_, the event rates (of both ER visits and admissions) were significantly higher in the abnormal group than in the normal group at all four areas (all *p* < 0.01).

## 4. Discussion

We found that abnormal PM_2.5_ concentrations measured outdoors, in the living room, and in the kitchen were linked to increased wheezing symptoms among patients with moderate to very severe COPD, similar to the findings of Hansel et al. [27], but different from those of Kaji et al. [28]. Kaji et al. reported that an increase of 10 μg/m^3^ of PM_2.5_ was associated with an increase of dyspnea (Modified Medical Research Council score), cough, and phlegm [28]. We also found higher abnormal outdoor PM_2.5_ concentrations were associated with higher CAT physical scores, indicating increased fine PM concentrations might be associated with worse respiratory symptoms in general. Higher abnormal PM_10_ concentrations measured in the living room and in the kitchen were associated with increased wheezing symptoms and reduced ADL.

Our results showed no association between PM_2.5_ and PM_10_ concentrations in the bedroom and respiratory symptoms. One possibility is that there were lower abnormal rates and concentrations of PM_2.5_ and PM_10_ in the bedroom. A 2014 study on sources of PM_2.5_ and outdoor air in Santiago, Chile found that the two main sources of PM_2.5_ were emissions of traffic and cooking at home [29]. Therefore, another reason may be that the two main sources of PM_2.5_ in the living room were emissions of traffic (outdoor) and cooking at home (kitchen), resulting in different PM characteristics between the bedroom and other areas. Moreover, our sample size may have been too small. We also found that outdoor PM_2.5_ concentrations were associated with wheezing, but did not find an association between outdoor PM_10_ and wheezing. The reason for this difference was the different particle size. For PM_2.5_, long residence times and high indoor–outdoor penetration efficiencies resulted in indoor–outdoor associations. However, PM_10_ had high deposition rates and low penetration efficiencies, meaning that outdoor PM_10_ may not be able to penetrate indoors [30].

We also found that higher abnormal PM_10_ concentrations increased the risk of AE of COPD. These data suggest that higher PM concentration levels were related to worse COPD health, and COPD patients may be susceptible to the effect of PM. Recent epidemiological evidence showed that ambient PM is directly associated with mortality of COPD [4], as well as with AE of COPD [5,6,7,8]. Few studies have focused on examining the effect of indoor air quality on COPD patients, such as quality of life and lung function [27,28,31,32,33,34]. A study including 148 patients with severe COPD reported that the levels of PM_2.5_ were associated with a worse quality of life [31]. Other studies, including 35 and 17 patients with severe COPD, showed no association between PM_2.5_ and PM_2.5–10_ and lung function [33,34]. In addition to assessing the relationship between indoor air quality and lung function, Hansel et al. investigated the health effects of PM_2.5_ on COPD patients using comprehensive clinical assessments, including lung function, respiratory symptoms, quality of life, and exacerbations [27]. The authors reported that higher levels of PM_2.5_ were associated with increased respiratory symptoms, which is consistent with our present findings. We did not find an association between PM_2.5_ and AE of COPD after controlling for lung function at each visit, age at baseline, smoking status, and whether the window was open or not. However, again, our sample size may have been too small. In addition, Xu et al. (2016) [35] reported a strong association between increases in PM concentration on the day prior to or on the third day after emergency room visits for COPD patients. The use of categorical data (normal vs. abnormal) might be another possible explanation for our different findings.

Our study has some limitations. First, our study only included 19 COPD patients and 83 observations. It may not be generalizable to the COPD population at large, and further well-powered studies are needed to obtain more precise findings. Second, the level of indoor PM varies depending on who is living in the home and on visitor characteristics and/or behaviors [29]. We did not use a personal exposure device to measure PM. We only measured PM concentrations in a short period of time, which may not represent long-term exposure. We also did not analyze the composition of PM. Future studies should analyze the composition of PM in order to identify the source of air pollution.

## 5. Conclusions

Our study shows that elevated PM concentrations are associated with worse respiratory symptoms and increased risk of COPD exacerbations in patients with moderate to very severe COPD. With only 19 patients in the study, conclusions might be not warranted. To our knowledge, this is the first longitudinal study to investigate the adverse respiratory health effects of exposure to indoor PM on COPD patients in Taiwan. Future studies are needed to determine the effectiveness of environmental interventions or self-management programs to reduce PM concentrations and improve health outcomes in this susceptible population.

## Figures and Tables

**Table 1:** Demographic and clinical characteristics of the study participants (*n* = 19).

Variable	*n* (%) or Mean ± Standard Deviation (SD)
Gender	
Male	19 (100.0)
Weight status	
Underweight	5 (26.3)
Normal	8 (42.1)
Overweight	3 (15.8)
Obese	3 (15.8)
Employment status	
Part-time job	2 (10.5)
Retired	17 (89.5)
Education level	
Uneducated	3 (15.8)
Elementary school	10 (52.6)
Junior high school or above	6 (31.6)
Regular exercise	
No	14 (73.7)
Yes	5 (26.3)
Cigarette smoking	
Quit	14 (73.7)
Current smoker	5 (26.3)
Family smoking	
No	10 (52.6)
Yes	8 (42.1)
Sometimes	1 (5.3)
Whether the window was open or not	
Always open	10 (52.6)
Always closed	8 (42.1)
Sometimes	1 (5.3)
Disease severity *	
Moderate	6 (31.6)
Severe	8 (42.1)
Very severe	5 (26.3)
Age (years)	72.6 ± 6.8
Body mass index (BMI) (kg/m^2^)	22.7 ± 6.7
Waist-hip ratio	1.00 ± 0.11
Charlson comorbidity index (CCI) ^#^	5.7 ± 1.1
FEV_1_ at 1st visit (%)	42.4 ± 15.0
Chronic obstructive pulmonary disease (COPD) duration (years)	5.1 ± 2.9

Note: * Based on guideline of Global Initiative for Chronic Obstructive Lung Disease (GOLD), moderate (79% ≤ FEV_1 predicted_ ≤ 50%); severe (49% ≤ FEV_1 predicted_ ≤ 30%); very severe (FEV_1 predicted_ < 30%). ^#^ Higher CCI scores indicate a higher level of burden of disease.

**Table 2:** Association between PM_2.5_ level and the symptoms and activities of daily living (ADL) of chronic obstructive pulmonary disease (COPD) patients.

Variable	Outdoor			Living Room			Bedroom			Kitchen		
	Normal ^#^ (*obs* = 6)	Abnormal * (*obs* = 77)	*B*	Normal ^#^ (*obs* = 9)	Abnormal * (*obs* = 74)	*B*	Normal ^#^ (*obs* = 13)	Abnormal * (*obs* = 70)	*B*	Normal ^#^ (*obs* = 7)	Abnormal * (*obs* = 76)	*B*
Respiratory Symptoms												
Cough	3.8 ± 1.8	3.3 ± 1.8	−0.20	3.8 ± 1.9	3.3 ± 1.8	−0.56	3.4 ± 1.9	3.3 ± 1.8	−0.10	3.4 ± 2.0	3.3 ± 1.8	−0.57
Phlegm	4.2 ± 1.3	3.5 ± 1.8	−0.22	3.8 ± 1.9	3.5 ± 1.8	−0.49	3.5 ± 1.8	3.5 ± 1.8	−0.12	3.7 ± 1.7	3.5 ± 1.8	−0.64
Dyspnea	2.2 ± 1.6	2.9 ± 1.6	0.84	3.1 ± 1.9	2.8 ± 1.6	−0.32	2.8 ± 1.8	2.8 ± 1.6	−0.10	2.0 ± 1.5	2.9 ± 1.6	0.48
Wheezing	1.2 ± 0.4	2.3 ± 1.5	1.46 **	1.1 ± 0.3	2.4 ± 1.6	0.80 ***	1.5 ± 1.2	2.4 ± 1.5	0.45	1.1 ± 0.4	2.3 ± 1.5	1.03 **
Total	11.3 ± 4.1	12.0 ± 4.8	1.88	11.8 ± 4.5	11.9 ± 4.8	−0.62	11.3 ± 5.3	12.0 ± 4.6	0.09	10.3 ± 4.6	12.1 ± 4.7	0.00
COPD Assessment Test (CAT)												
Physical	7.2 ± 4.0	7.8 ± 4.2	0.93 *	8.1 ± 4.7	7.8 ± 4.2	−0.16	8.3 ± 5.0	7.7 ± 4.1	−0.82	5.7 ± 4.0	8.0 ± 4.2	0.16
Psycho	2.7 ± 2.0	3.1 ± 2.7	0.04	2.6 ± 2.0	3.1 ± 2.7	0.61	2.8 ± 2.0	3.1 ± 2.7	0.12	1.7 ± 1.7	3.2 ± 2.7	0.15
Total	9.8 ± 5.9	10.9 ± 6.5	0.93	10.8 ± 6.7	10.9 ± 6.5	0.34	11.2 ± 7.0	10.8 ± 6.4	−0.81	7.4 ± 5.7	11.2 ± 6.5	0.23
London ADL												
Self-care	21.0 ± 6.0	20.3 ± 4.8	0.17	18.1 ± 7.5	20.6 ± 4.4	2.15	18.5 ± 6.5	20.7 ± 4.5	1.98	21.9 ± 5.8	20.2 ± 4.8	0.69
Domestic	22.3 ± 17.3	19.6 ± 13.7	−1.28	17.6 ± 13.2	20.1 ± 14.0	4.26	18.0 ± 14.6	20.1 ± 13.8	3.77	21.7 ± 15.0	19.6 ± 13.8	2.19
Sport	3.2 ± 2.7	4.0 ± 2.4	0.38	4.2 ± 3.7	3.9 ± 2.2	−0.53	4.4 ± 3.5	3.9 ± 2.2	−0.83	2.4 ± 2.7	4.1 ± 2.3	0.07
Leisure	1.5 ± 3.2	1.4 ± 1.8	−0.15	2.0 ± 2.8	1.4 ± 1.8	−0.56	1.8 ± 2.4	1.4 ± 1.8	−0.32	1.3 ± 3.0	1.4 ± 1.8	−0.34
Total	48.0 ± 17.5	45.3 ± 13.9	−0.84	41.9 ± 14.1	46.0 ± 14.1	5.27	42.7 ± 15.0	46.1 ± 13.9	4.55	47.3 ± 15.4	45.4 ± 14.0	2.63

Note: *B* = regression coefficient derived from the generalized estimating equation (GEE) model with adjustment for age at baseline, forced expiratory volume in 1 second (FEV_1_) predicted at each visit, smoking status, and whether the window was open or not; *obs* = observation; ^#^ Normal: Daily mean or 24-h maximum of PM_10_ is below 125 μg/m^3^, and that of PM_2.5_ is below 35 μg/m^3^; * Abnormal: Daily mean or 24-h maximum of PM_10_ is above 125 μg/m^3^, and that of PM_2.5_ is above of 35 μg/m^3^; * *p* < 0.05; ** *p* < 0.01; *** *p* < 0.001.

**Table 3:** Association between PM_10_ and the symptoms and activities of daily living of COPD patients.

Variable	Outdoor			Living Room			Bedroom			Kitchen		
	Normal ^#^ (*obs* = 43)	Abnormal * (*obs* = 40)	*B*	Normal ^#^ (*obs* = 46)	Abnormal * (*obs* = 37)	*B*	Normal ^#^ (*obs* = 50)	Abnormal * (*obs* = 33)	*B*	Normal ^#^ (*obs* = 46)	Abnormal * (*obs* = 37)	*B*
Respiratory Symptoms												
Cough	3.3 ± 1.8	3.4 ± 1.9	−0.25	3.4 ± 1.8	3.2 ± 1.8	−0.26	3.5 ± 1.8	3.2 ± 1.9	−0.08	3.3 ± 1.8	3.4 ± 1.8	−0.06
Phlegm	3.4 ± 1.7	3.6 ± 1.8	−0.07	3.5 ± 1.7	3.5 ± 1.8	−0.24	3.6 ± 1.7	3.4 ± 1.9	0.04	3.4 ± 1.7	3.7 ± 1.8	0.02
Dyspnea	2.6 ± 1.6	3.1 ± 1.6	0.40	2.7 ± 1.7	3.0 ± 1.5	0.39	2.8 ± 1.7	2.8 ± 1.5	0.13	2.6 ± 1.7	3.1 ± 1.5	0.25
Wheezing	2.1 ± 1.5	2.4 ± 1.6	0.43	2.0 ± 1.5	2.5 ± 1.5	0.36 *	2.1 ± 1.6	2.4 ± 1.5	0.18	2.0 ± 1.5	2.5 ± 1.5	0.38 *
Total	11.4 ± 5.1	12.5 ± 4.2	0.40	11.6 ± 5.0	12.3 ± 4.3	0.30	12.0 ± 4.9	11.8 ± 4.5	0.32	11.3 ± 5.2	12.7 ± 4.0	0.51
CAT												
physical	7.6 ± 4.4	8.0 ± 4.1	−0.16	7.5 ± 4.3	8.2 ± 4.1	0.29	7.8 ± 4.3	7.8 ± 4.1	0.67	7.2 ± 4.2	8.5 ± 4.1	0.47
psycho	2.7 ± 2.4	3.4 ± 2.8	0.20	2.7 ± 2.4	3.4 ± 2.9	0.41	2.9 ± 2.5	3.2 ± 2.8	0.10	2.5 ± 2.2	3.7 ± 3.0	0.67
Total	10.3 ± 6.5	11.4 ± 6.5	0.00	10.2 ± 6.3	11.6 ± 6.6	0.65	10.7 ± 6.5	11.1 ± 6.5	0.75	9.8 ± 6.1	12.2 ± 6.7	1.10
London ADL												
Self-care	20.3 ± 5.3	20.4 ± 4.4	0.67	20.2 ± 5.1	20.6 ± 4.5	0.49	19.8 ± 5.3	21.2 ± 3.9	1.27	20.5 ± 5.1	20.2 ± 4.5	0.20
Domestic	18.8 ± 15.2	20.8 ± 12.3	3.28	18.3 ± 15.0	21.7 ± 12.2	4.07	19.0 ± 14.2	21.0 ± 13.5	4.15	17.8 ± 15.3	20.2 ± 11.5	4.95
Sport	3.8 ± 2.6	4.1 ± 2.1	−0.09	3.9 ± 2.5	4.0 ± 2.2	−0.32	4.1 ± 2.5	3.7 ± 2.2	−0.69	3.8 ± 2.5	4.1 ± 2.2	−0.22
Leisure	1.3 ± 2.1	1.6 ± 1.8	0.32	1.3 ± 2.0	1.6 ± 1.8	0.58	1.4 ± 2.1	1.5 ± 1.6	0.28	1.2 ± 2.0	1.6 ± 1.8	0.52
Total	44.3 ± 15.4	46.9 ± 12.5	4.09	43.6 ± 14.8	48.9 ± 12.9	5.58 *	44.3 ± 14.1	47.4 ± 13.9	5.34	43.4 ± 15.1	48.2 ± 12.4	6.18 *

Note: *B* = regression coefficient derived from the GEE model with adjustment for age at baseline, FEV_1_ predicted at each visit, smoking status, and whether the window was open or not; *obs* = observation; ^#^ Normal: Daily mean or 24-h maximum of PM_10_ is below 125 μg/m^3^, and that of PM_2.5_ is below 35 μg/m^3^; * Abnormal: Daily mean or 24-h maximum of PM_10_ is above 125 μg/m^3^, and that of PM_2.5_ is above of 35 μg/m^3^; * *p* < 0.05.

**Table 4:** Association between particulate matter (PM_2.5_ and PM_10_) and acute exacerbation in COPD patients.

Parameter	No. of *obs*	Emergency Room (ER) Visit			Admission Due to Acute Exacerbation (AE)		
		Event (%)	OR (95% CI)	*p*	Event (%)	OR (95% CI)	*p*
PM_2.5_							
Outdoor							
Normal ^#^	6	0 (0.0)	Reference	-	0 (0.0)	Reference	-
Abnormal *	77	26 (33.8)	NA	NA	26 (33.8)	NA	NA
Living room							
Normal ^#^	9	0 (0.0)	Reference	-	0 (0.0)	Reference	-
Abnormal *	74	26 (35.1)	NA	NA	26 (35.1)	NA	NA
Bedroom							
Normal ^#^	13	0 (0.0)	Reference	-	0 (0.0)	Reference	-
Abnormal *	70	26 (37.1)	NA	NA	26 (37.1)	NA	NA
Kitchen							
Normal ^#^	7	0 (0.0)	Reference	-	0 (0.0)	Reference	-
Abnormal *	76	26 (34.2)	NA	NA	26 (34.2)	NA	NA
PM_10_							
Outdoor							
Normal ^#^	43	3 (7.0)	Reference	-	3 (7.0)	Reference	-
Abnormal *	40	23 (57.5)	30.1 (4.9–184.2)	<0.001	23 (57.5)	19.5 (4.7–80.6)	<0.001
Living room							
Normal ^#^	46	4 (8.7)	Reference	-	5 (10.9)	Reference	-
Abnormal *	37	22 (59.5)	23.8 (3.0–191.3)	0.003	21 (56.8)	16.2 (3.1–84.9)	0.001
Bedroom							
Normal ^#^	50	7 (14.0)	Reference	-	7 (14.0)	Reference	-
Abnormal *	33	19 (57.6)	12.1 (2.5–60.0)	0.002	19 (57.6)	10.5 (2.5–44.6)	0.001
Kitchen							
Normal ^#^	46	3 (6.5)	Reference	-	4 (8.7)	Reference	-
Abnormal *	37	23 (62.2)	38.5 (4.8–311.8)	0.001	22 (59.5)	18.5 (3.7–91.9)	<0.001

Note: OR = odds ratio derived from GEE model with adjustment of age at baseline, FEV_1_ predicted at each visit, smoking status, and CI = confidence interval; *obs* = observation; ^#^ Normal: Daily mean or 24-h maximum of PM_10_ is below 125 μg/m^3^, and that of PM_2.5_ is below 35 μg/m^3^; * Abnormal: Daily mean or 24-h maximum of PM_10_ is above 125 μg/m^3^, and that of PM_2.5_ is above of 35 μg/m^3^.
